# Altered Myokine Secretion Is an Intrinsic Property of Skeletal Muscle in Type 2 Diabetes

**DOI:** 10.1371/journal.pone.0158209

**Published:** 2016-07-25

**Authors:** Theodore P. Ciaraldi, Alexander J. Ryan, Sunder R. Mudaliar, Robert R. Henry

**Affiliations:** 1 Veterans Affairs San Diego Healthcare System, San Diego, CA, United States of America; 2 Department of Medicine, Division of Endocrinology & Metabolism, University of California San Diego, La Jolla, CA, United States of America; University of Catanzaro Magna Graecia, ITALY

## Abstract

Skeletal muscle secretes factors, termed myokines. We employed differentiated human skeletal muscle cells (hSMC) cultured from Type 2 diabetic (T2D) and non-diabetic (ND) subjects to investigate the impact of T2D on myokine secretion. Following 24 hours of culture concentrations of selected myokines were determined to range over 4 orders of magnitude. T2D hSMC released increased amounts of IL6, IL8, IL15, TNFa, Growth Related Oncogene (GRO)a, monocyte chemotactic protein (MCP)-1, and follistatin compared to ND myotubes. T2D and ND hSMC secreted similar levels of IL1ß and vascular endothelial growth factor (VEGF). Treatment with the inflammatory agents lipopolysaccharide (LPS) or palmitate augmented the secretion of many myokines including: GROa, IL6, IL8, IL15, and TNFa, but did not consistently alter the protein content and/or phosphorylation of IkBa, p44/42 MAPK, p38 MAPK, c-Jun N-terminal kinase (JNK) and NF-kB, nor lead to consistent changes in basal and insulin-stimulated glucose uptake or free fatty acid oxidation. Conversely, treatment with pioglitazone or oleate resulted in modest reductions in the secretion of several myokines. Our results demonstrate that altered secretion of a number of myokines is an intrinsic property of skeletal muscle in T2D, suggesting a putative role of myokines in the response of skeletal muscle to T2D.

## Introduction

As the primary tissue responsible for post-prandial glucose disposal [1], skeletal muscle plays a key role in regulating metabolism. Skeletal muscle insulin resistance is an early event in the development of Type 2 Diabetes (T2D) [2]. Aside from its roles in locomotion and metabolism, skeletal muscle has been shown to act as an endocrine tissue, with the capacity to synthesize and secrete multiple factors, now referred to as ‘myokines’ [3]. A major advance in this area was the observation of Pedersen and colleagues that increases in circulating IL6 following exercise resulted from increases in skeletal muscle IL-6 synthesis and secretion [4]. Since those early findings an increasing number of other myokines have been identified [5]. Although many of these factors, including cytokines and chemokines are produced by multiple cell types [6], some appear to be more exclusive to muscle, such as myostatin [7].

Focusing on studies in human muscle, the synthesis and secretion of myokines is regulated by a number of conditions including; differentiation [8], exercise [5], electrical stimulation in vitro [9, 10] and induction of insulin resistance [11]. Studies of the impact of T2D on myokine production are limited. Cultured myotubes from T2D subjects displayed elevated mRNA content for TNFa and MCP-1 compared to cells from non-diabetic (ND) individuals [12]; we reported previously that release of TNFa protein was also elevated [13]. Meanwhile, IL6 mRNA and secretion did not differ between T2D and ND myotubes [12, 14]. However, caution must be exerted while determining the significance of changes in mRNA content, as there are multiple examples of discrepancies between mRNA content and expression and/or secretion of the same protein [15].

Studies performed in primary or cultured myotubes can provide specific information about the regulation of myokine synthesis and secretion. Human skeletal muscle satellite cells cultured and differentiated to myotubes (hSMC) have been validated as a system that retains many of the properties of muscle studied in vivo [16]. Hence, myotubes from T2D subjects display impairments in glucose uptake [17], glycogen synthesis [18] and fatty acid oxidation [19, 20]. These properties are present even when hSMC are removed from the hyperglycemic and hyperinsulinemic environment characteristic of T2D. In the current report we examined the secretion and regulation of a number of potential myokines by hSMC obtained from ND and T2D subjects, finding that the secretory profiles of ND and T2D myotubes differ in a number of potentially important ways.

## Materials and Methods

### Materials

Cell culture materials were purchased from Irvine Scientific (Irvine, CA) or GIBCO (Grand Island, NY) except for skeletal muscle growth medium, which was obtained from Lonza (Walkersville, MD). [3H] 2-deoxyglucose, [^14^C]-L-glucose and [^3^H]-palmitate were obtained from Perkin Elmer (Boston, MA). All other chemicals were reagent grade and purchased from Sigma Chemical (St. Louis, MO), except for AG-1X8 ion exchange resin (Bio-Rad, Richmond, CA). Electrophoresis reagents were from Bio-Rad or Invitrogen (Carlsbad, CA). Primary antibodies were obtained from the following sources: IkBa (catalog #9242), phospho-p44/42 MAPK (#9106), p44/42 MAPK (#4695), phospho-p38 MAPK (#9216), p38 MAPK (#9212), phospho-NF-kB p65 (#13346), NF-kB p65 (#8242) (Cell Signaling Technology, Beverly, MA), phospho-JNK (#sc-6254), JNK (#sc-571), MyoD (#sc-760) (Santa Cruz Biotechnology, Santa Cruz, CA), b-actin (#NB600-503) (Novus, Littleton, CO). Fluorescently labeled secondary antibodies and blocking buffer were obtained from Licor (Lincoln, NE).

### Subjects

Muscle biopsy samples were obtained from 26 ND subjects and 21 T2D subjects. General inclusion criteria included: weight stable (± 2 kg) for 1 month and medication use stable for at least 3 months. Use of steroids and anti-depressants were cause for exclusion. Criteria for classification as ND were [fasting glucose] < 100 mg/dL at the screening visit and HbA1c <5.7% within 2 weeks of biopsy. None of the subjects from the ND group had a family history of type 2 diabetes and none were taking medications that influenced glucose metabolism. All of the women studied were post-menopausal (6–13 yr for ND, 6–11 yr for T2D), obviating the need to take into account phase of the menstrual cycle for the timing of sample collection. None of the women were taking hormonal replacement therapy. The experimental protocol was approved by the Human Research Protection Programs of the San Diego VA Healthcare System and the University of California, San Diego. Informed written consent was obtained from all subjects after explanation of the protocol.

Blood was collected after an overnight (10–12 hr) fast, serum prepared and stored at -80°C before analysis. Percutaneous needle biopsies of vastus lateralis muscle were performed and muscle tissue was immediately processed for culture as previously described [21].

### Skeletal muscle cell culture, treatment and generation of conditioned media

The techniques of muscle satellite cell isolation and growth have been described in detail previously, including the fact that the extent of differentiation into myotubes was similar in cells from ND and T2D subjects [21]. Cells were used after the first passage. After attaining 80–90% confluence, cells were fused for 5 days in α-MEM containing 2% fetal bovine serum (FBS), 100 U/ml penicillin and 100 mg/ml streptomycin. After three washes with PBS, the media was replaced with serum-free α-MEM (0.1% BSA) containing antibiotics and the indicated treatments. Final vehicle (DMSO or EtOH) concentrations did not exceed 0.05%. Oleic acid was purchased already complexed with BSA (Sigma). Palmitate was conjugated to fatty acid-free BSA by the method described by Sinha et al [22]. Media was collected after 24 hours. Parallel flasks and plates were treated for 48 hr before media collection and cell protein extraction, glucose uptake, and fatty acid oxidation. Media was centrifuged (800 x g, 10 min, 4°C) to remove debris, and stored at -80°C. Culture in serum-free α-MEM for up to 48 hr had no effect on cell viability, protein content, or extent of differentiation [23].

### Glucose uptake assay

Uptake of the non-metabolized analog 2-deoxyglucose (final concentration = 0.01 mM) was measured in triplicate over 10 minutes at room temperature [17]. An aliquot of the suspension was removed for protein analysis. The uptake of L-glucose was used to correct each sample for the contribution of diffusion. All results are adjusted for total cellular protein content determined by the method of Bradford [24].

### Free Fatty Acid (FFA) oxidation

Fatty acid metabolism was assayed by b-oxidation of the long chain fatty acid palmitate. Cells were incubated in serum-free α-MEM containing [9,10-^3^H] palmitic acid (final concentration = 20 μM) in a 95% O_2_:5% CO_2_ incubator at 37°C for 3h. The final reaction volume was 500 mL. After incubation, an aliquot (100 mL) of the culture medium was placed over an ion-exchange resin and the column washed with 1.5 mL of water. Intact FFA (charged state) was retained by the resin, whereas the oxidized portion of FFA passed freely through the column in the form of water. Results were adjusted for total cellular protein content.

### Protein extraction

Muscle cells were rapidly washed 5x with 4°C PBS and then lysed in extraction buffer [25]. Protein concentration was determined by the Bradford assay and extracts stored at -80°C until analyzed.

### Assay for circulating and secreted factors

Serum insulin levels were determined by RIA (Millipore Corp, Billerica, MA), with intra-assay coefficient of variation (CV) less than 7%. The majority of myokines in serum and conditioned media were analyzed with MILLIPLEX MAP kits (Millipore) using a BioPlex 200 instrument (Bio-Rad Corp, Hercules, CA). Sensitivities (in pg/mL), inter- and intra-assay CVs for each analyte are as follows: IL1b (0.4, 7%, 6%), IL6 (0.3, 12, 8), IL8 (0.2, 12, 7), IL10 (0.3, 9, 5), IL15 (0.4, 10, 7), GROa (10.1, 12, 5), TNFa (0.1, 16, 10), interferon-g (IFNg) (0.1, 6, 5), MCP-1 (0.9, 12, 6), VEGF (5.8, 8, 6). Follistatin was measured by ELISA (R&D Systems, Minneapolis, MN) (83, 6.4, 2.5).

### Electrophoresis and Western blotting

Procedures for the electrophoresis, transfer and western blotting of proteins are similar to standard methods. For analysis of protein phosphorylation, membranes were probed with antibodies directed against phospho- and total protein produced in different species. Equal amounts of protein were loaded in each lane. Uniformity of loading was monitored by blotting for b-actin. A sample of human skeletal muscle tissue protein was included on each gel to serve as a control for inter-gel variability. Detection and quantification of band intensity was performed using a LiCor Odyssey CxLsystem with ImageStudio analysis software (v.3.1.4), with results normalized against the loading control.

### Statistical analysis

Statistical analysis was performed using GraphPad Prism 5.0 (GraphPad, San Diego, CA) and the Statistical Package for Social Sciences v.19 (SPSS, Chicago, IL). Between group comparisons were evaluated by independent group *t* test if data was normally distributed and with a Mann-Whitney test for non-normally distributed data, and by ANCOVA after adjusting for age and body mass index (BMI). Within group comparisons (treatment effects) were evaluated by paired *t* test of absolute values. For results that were not normally distributed, data was log-transformed for statistical analysis and then back-transformed and reported in original units as mean ± SEM. The Pearson correlation test was used for univariate correlation analysis. Statistical significance was accepted as p<0.05. The number of individual determinations for each measurement is indicated in the Fig legends.

## Results

### Subjects and circulating cytokine and chemokine levels

Subject characteristics are presented in Table 1. The designation of T2D was made on the basis of an existing clinical diagnosis, including [HbA1c] = 7.5–9.5%. Duration of diabetes ranged from 1–18 years. Medication use was stable for at least 3 months before biopsy; all T2D subjects remained on their prescribed medications up to the day of biopsy. Anti-diabetic medication use included: metformin alone (n = 9), metformin + glipizide (n = 4), metformin + glyburide (n = 2), metformin + glargine (n = 2), glipizide alone (n = 1). Three subjects were controlled without medication. There was a tendency for T2D subjects to be older (p = 0.054). The T2D subjects were more overweight-to-obese than the ND individuals, and displayed a high degree of insulin resistance in the fasting state. Circulating levels of a number of cytokines and chemokines, some of which have previously been validated as myokines, such as IL6, TNFa and MCP-1 (3),were evaluated in the fasting state. While considerable variability was present, levels of TNFa, GROa and follistatin were found to be higher in the T2D subjects (Table 2). However, the diabetes-related differences in TNFa and GROa were lost after adjusting for BMI.

**Table 1:** Subject Characteristics.

Group	Non-diabetic	Type 2 diabetes
n (F/M)	26 (4/22)	21 (5/16)
Age (yrs)	51 ± 2	57 ± 2
BMI (kg/m^2^)	28.6 ± 0.8	32.9 ± 1.3†
Fasting [glucose] (mM)	5.09 ± 0.10	9.19 ± 0.84†
Fasting [insulin] (pM)	43 ± 8	127 ± 26†
HOMA-IR	1.28 ± 0.29	4.95 ± 0.87†

† p<0.01 vs ND, not adjusted for age or BMI.

All differences remained statistically significant after adjusting for age and/or BMI.

**Table 2:** Circulating cyto- and chemokine levels.

[factor] (pg/mL)	ND	T2D
IL-1b	1.96 ± 0.56	1.68 ± 0.65
IL-6	4.56 ± 2.54	3.07 ± 1.17
IL-8	7.88± 1.02	10.26 ± 1.31
IL-10	5.23 ± 1.01	7.89 ± 2.22
IL-15	3.80 ± 0.50	5.72 ± 1.89
IFNg	6.79 ± 3.64	5.92 ± 1.87
TNFa	3.99 ± 0.36	6.16 ± 0.52*
MCP1	303 ± 26	277 ± 31
MIP1a	2.61 ± 1.00	8.09 ± 5.18
GROa	472 ± 88	885 ± 193*
VEGF	97.5 ± 16.4	82.6 ± 26.5
Follistatin	1366 ± 153	2159 ± 327*

*p<0.05 vs ND, not adjusted for age or BMI.

### Myokine secretion

In order to evaluate the impact of T2D on myokine secretion, we employed the hSMC system on which we have published extensively over the past two decades. Fully differentiated myotubes cultured from subjects with T2D displayed impairments in basal (11.57 ± 1.26 vs 18.48 ± 2.51 pmol/mg protein/min, T2D vs ND, p<0.05) and insulin-stimulated (15.52 ± 1.72 vs 20.76 ± 2.71, p = 0.10) glucose uptake, as well as b-oxidation of palmitate (14.05 ± 4.85 vs 35.36 ± 8.78 nmol/mg protein, p<0.05), similar to what we have reported previously [19, 21].

**Fig 1:**
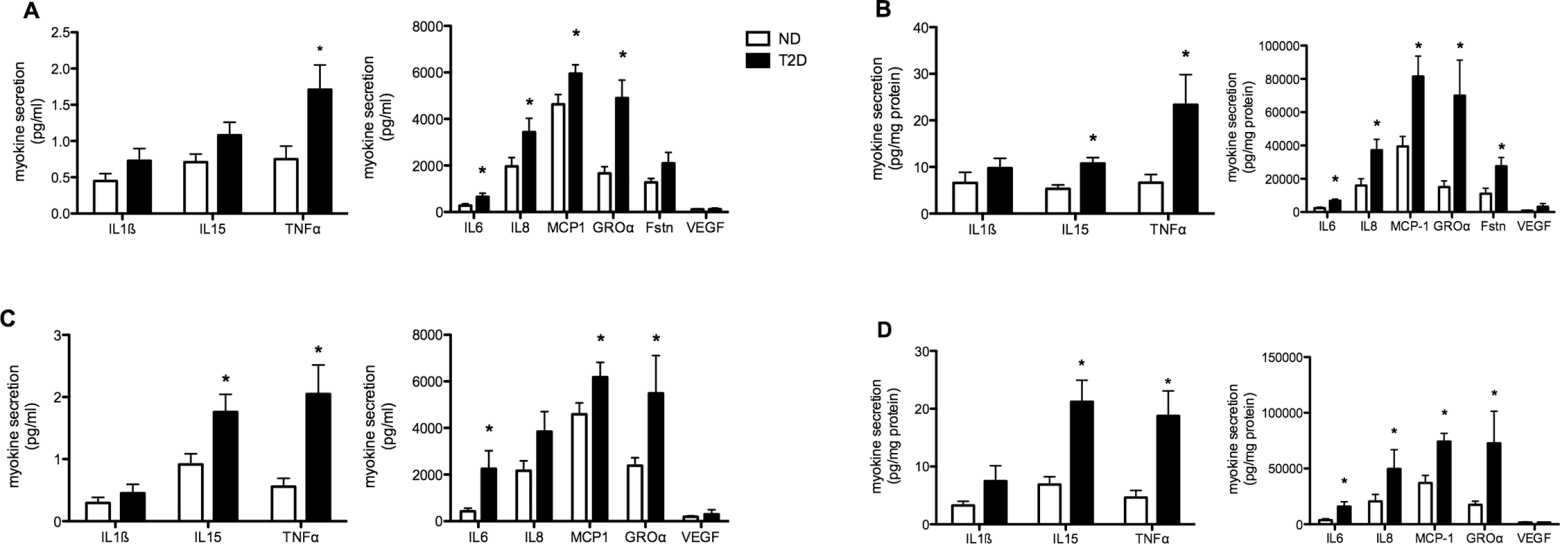
Myokine secretion from differentiated hSMC obtained from non-diabetic (ND, open bars) and Type 2 Diabetic (T2D, solid bars) subjects. (A) Media concentrations of selected factors after 24 hr of culture. (B) 24 hr secretion normalized against total cell protein for each individual set of cells. N = 8–23 for ND, 8–18 for T2D. (C) Media concentrations after 48 hr in culture. (D) 48 hr secretion normalized against cell protein. n = 8–18 for ND, 5–14 for T2D. Results are average + SEM. *p<0.05 vs ND

Myotube conditioned media was collected after 0–24 and 0–48 hours in culture and the release of selected cyto- and chemokines, hereafter referred to as ‘myokines’, was measured. In deciding which factors to measure, we elected to follow several that had already been investigated in human myotubes (IL6, TNFa, MCP-1) and others that had the potential to influence metabolism and/or inflammation. During the first 24 hours levels of secreted myokines ranged over four orders of magnitude, with MCP-1, IL8, GROa and follistatin amongst the most abundant factors (Fig 1A). T2D myotubes released significantly higher amounts of IL6, IL8, MCP-1, TNFa and GROa compared to ND cells, with a strong tendency for IL15 (p = 0.056) release to also be elevated. After 48 hours in culture, secretion of IL15 was significantly higher from T2D myotubes (Fig 1C). Even though all cultures were fully confluent, myotube protein content can vary considerably between individuals. To take this into account, myokine secretion was normalized against cell protein. When this adjustment was made, secretion of IL6, IL8, IL15, GROa, TNFa, MCP-1 and follistatin were all significantly higher from T2D myotubes, depending on the time evaluated (Fig 1B & 1D). ND and T2D myotubes secreted similar amounts of IL1b and VEGF. Of other factors of interest, IFNg in the media was consistently below the limit of detection, regardless of the time of culture, and IL10 levels often did not exceed the lower limit of detection; this was seen with both ND and T2D hSMC.

### Regulation of myokine secretion

**Fig 2:**
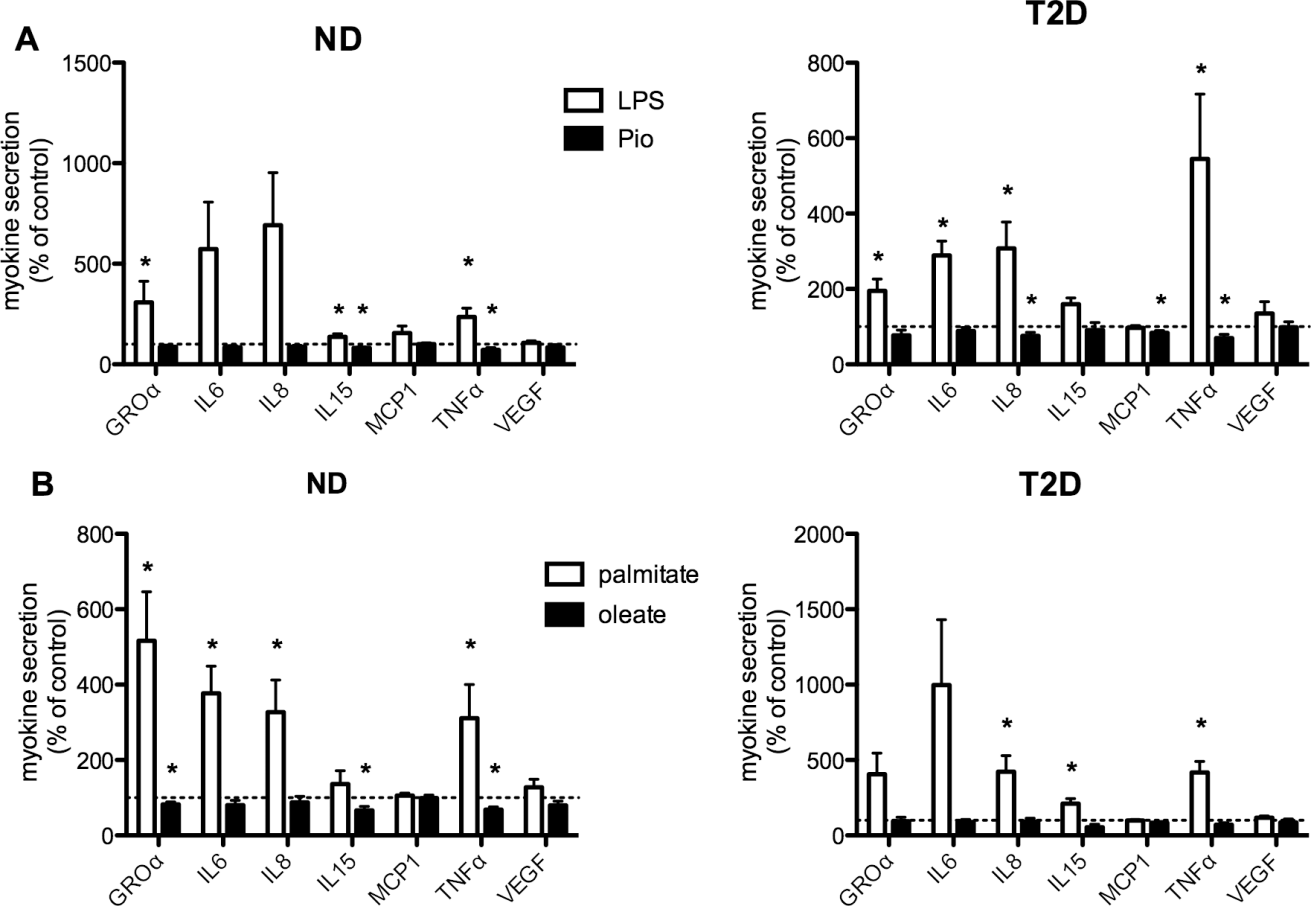
Regulation of myokine secretion. (A) Secretion over 24 hr with treatment by LPS (1 mg/mL, open bars) or Pio (10 mM, solid bars). n = 13–19 for ND, 8–21 for T2D. (B) Secretion over 24 hr with treatment by palmitate (0.3 mM, open bars) or oleate (0.3 mM, solid bars). Results are expressed relative to secretion in untreated (control, indicated by dashed line) cells from each individual set of cells, average + SEM, n = 7–10 for ND, 5–10 for T2D *p<0.05 vs paired control

Many of the myokines we found to be secreted to a greater extent by T2D myotubes are known pro-inflammatory cytokines and chemokines. Next, we investigated the impact of exposure to an inflammatory stimulus, lipopolysaccharide (LPS, 1 mg/mL) [26] on the secretion of selected myokines. While statistical analysis of treatment effects on myokine secretion was performed on absolute values, due to the wide range in absolute values, results are presented relative to the value in untreated (control) cells from each individual subject. Secretion of a number of myokines was increased over 24 hr of treatment with LPS (Fig 2A). In myotubes from ND subjects, LPS-induced changes in secretion attained statistical significance for GROa, IL15 and TNFa. In myotubes from T2D subjects, in addition to GROa, IL15, and TNFa, secretion of IL6 and IL8 was also stimulated to a significant degree by LPS treatment. LPS had no effect on secretion of follistatin (data not shown) or VEGF from either ND or T2D myotubes. Meanwhile, pioglitazone treatment significantly suppressed secretion of IL15 and TNFa from ND hSMC, and TNFa, IL8, and MCP-1 in T2D cells. Similar results were observed after 48 hours of treatment (data not shown).

Saturated fatty acids have also been shown to induce an inflammatory response in multiple cell types, including skeletal muscle [27]. We compared the effects on myokine secretion of exposure to equal concentrations (0.3 mM) of the pro-inflammatory saturated long chain fatty acid palmitate and the non-inflammatory mono-unsaturated oleate. In ND myotubes, 24 hr of palmitate treatment had a comparable or greater impact than treatment with LPS, leading to significant stimulations of GROa, IL6, IL8, and TNFa release (Fig 2B). Generally similar results were seen in T2D myotubes, though the responses attained statistical significance only for IL8, TNFa, and IL15. Treatment with oleate had effects generally comparable to that with Pio, at least in ND cells, as secretion of GROa, IL15 and TNFa was reduced. T2D myotubes appeared to be less responsive to oleate, as secretion of each of the myokines evaluated was unaltered (Fig 2B). It should be noted that none of the treatments had a consistent effect on either total cellular protein or extent of differentiation (see below).

### Relationship between regulation of myokine secretion and metabolism

**Fig 3:**
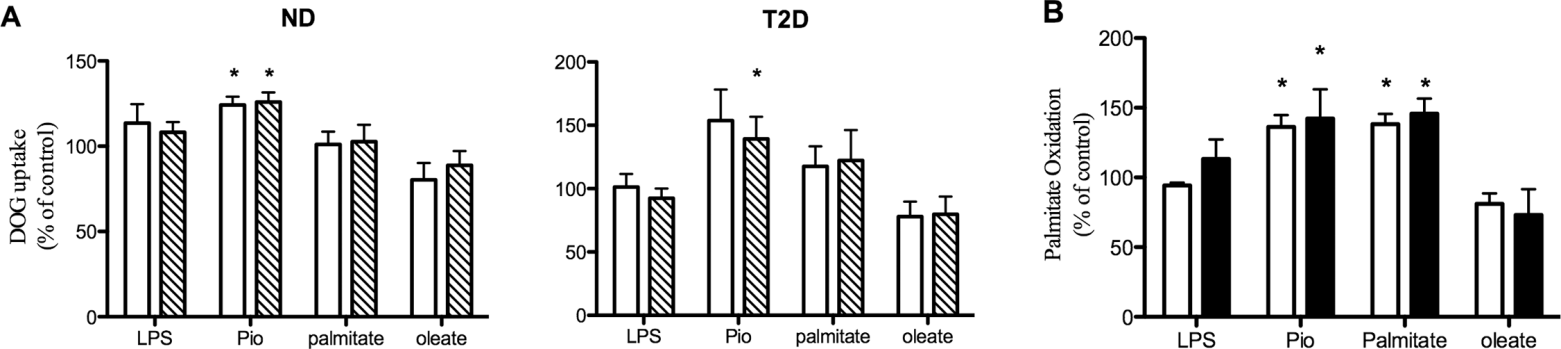
Regulation of metabolism by treatments that influence myokine secretion. Cells were treated as described in Fig 2, but for 48 hr before assay. (A) Basal (open bars) and insulin-stimulated (hatched bars, 33 nM, 60 min) 2-deoxyglucose uptake. Results expressed relative to untreated control, either basal or insulin-stimulated, in each individual set of cells, average + SEM, n = 9 for ND, 8–10 for T2D. (B) Palmitate oxidation in ND (open bars, n = 3) and T2D (solid bars, n = 5–6) hSMC. *p<0.05 vs paired control

The potential autocrine impact of changes in myokine secretion on myotube metabolism was investigated by assaying glucose uptake and b-oxidation of palmitate in hSMC treated for 2 days under the same conditions where myokine secretion was measured. It should be noted that this design does not distinguish between the direct effects of the specific treatment present in the CM or those of changes in myokine secretion. Results are presented normalized against the control (untreated) basal and insulin-stimulated glucose uptake and FFA oxidation activities for each individual; statistical analysis was performed on absolute activities. The increases in the secretion of multiple myokines after LPS and palmitate treatment (Fig 2) did not result in appreciable changes in either basal or insulin-stimulated glucose uptake; this lack of response was similar in ND and T2D hSMC (Fig 3A). Just as we have reported previously [28], Pio treatment led to upregulation of basal and insulin-stimulated glucose uptake in both groups (p = 0.053 for Pio on basal glucose uptake in T2D hSMC). There was a tendency for oleate to reduce basal glucose uptake in ND (p = 0.079) and T2D (p = 0.101) myotubes. While Pio and palmitate treatment had opposing effects on the secretion of multiple myokines (Fig 2), both resulted in significant stimulation of fatty acid ß-oxidation in ND hSMC (Fig 3B); the Pio effect in this cohort of T2D cells did not attain statistical significance (p = 0.115). Neither LPS nor oleate treatment caused significant changes in palmitate oxidation.

### Myokines and inflammatory signaling in myotubes

**Fig 4:**
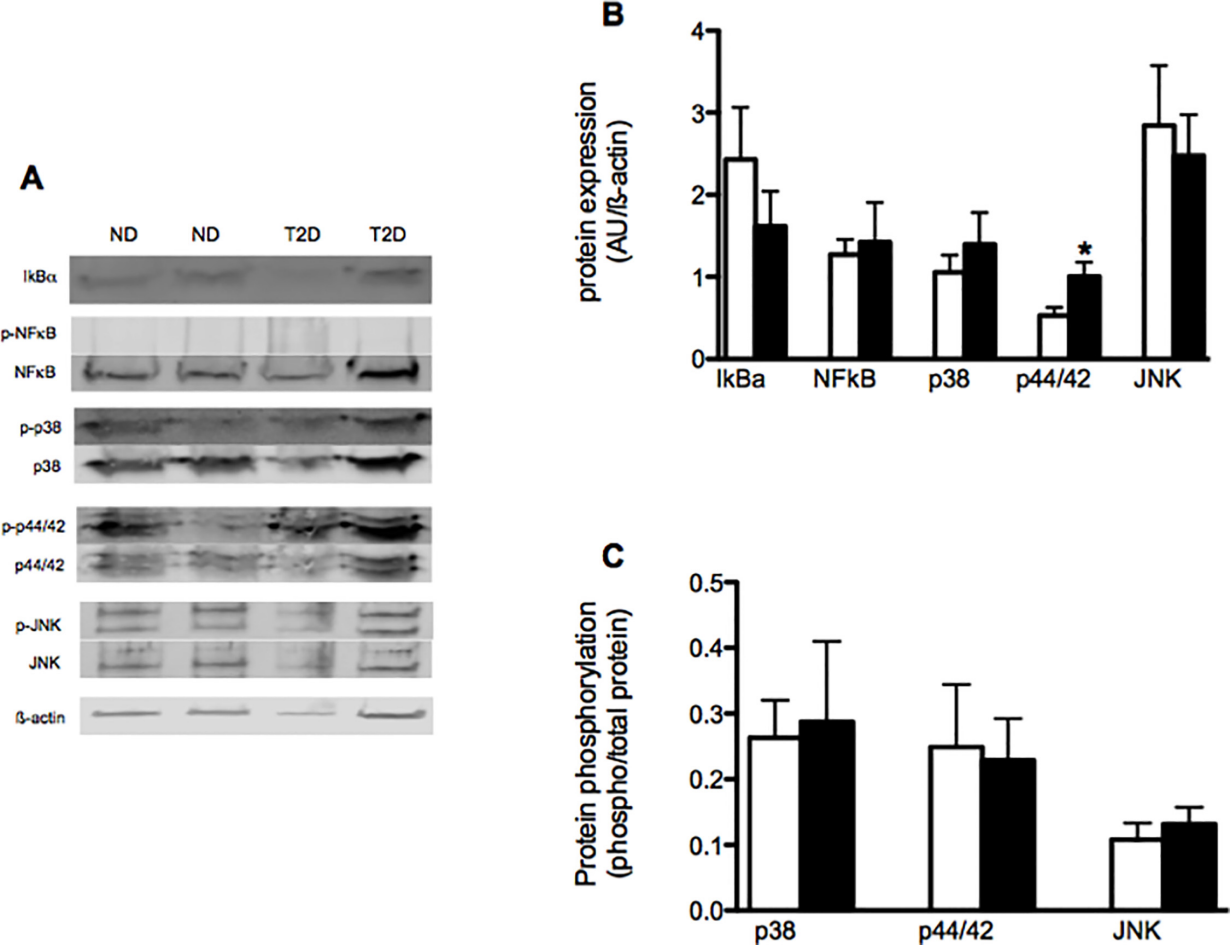
Impact of T2D on inflammatory signaling in hSMC. Proteins extracted from ND (open bars) and T2D (solid bars) hSMC under control conditions. (A) Representative western blots. (B) Quantification of protein expression normalized to loading control (ß-actin). Ave + SEM, n = 13–21 for ND, 10–16 for T2D. (C) Protein phosphorylation, expressed as ratio of phosphorylated to total protein. Ave + SEM, n = 13–17 for ND, 9–15 for T2D. *p<0.05 vs ND

The impact of prolonged exposure of T2D myotubes to elevated levels of pro-inflammatory myokines was evaluated by following the expression and phosphorylation of key proteins involved in classic inflammatory signaling. IkBa protein expression did not differ between ND and T2D cells under control conditions (Fig 4B). Expression of the p65 subunit of NFkB also did not differ between ND and T2D myotubes: NFkB phosphorylation was not detectable. The expression of p44/42 MAPK was elevated in T2D hSMC (Fig 4B), while phosphorylation (Fig 4C) was similar in ND and T2D cells. Meanwhile, the protein expression (Fig 4B) and phosphorylation (Fig 4C) of both p38 MAPK and JNK were similar between ND and T2D myotubes under control conditions.

**Fig 5:**
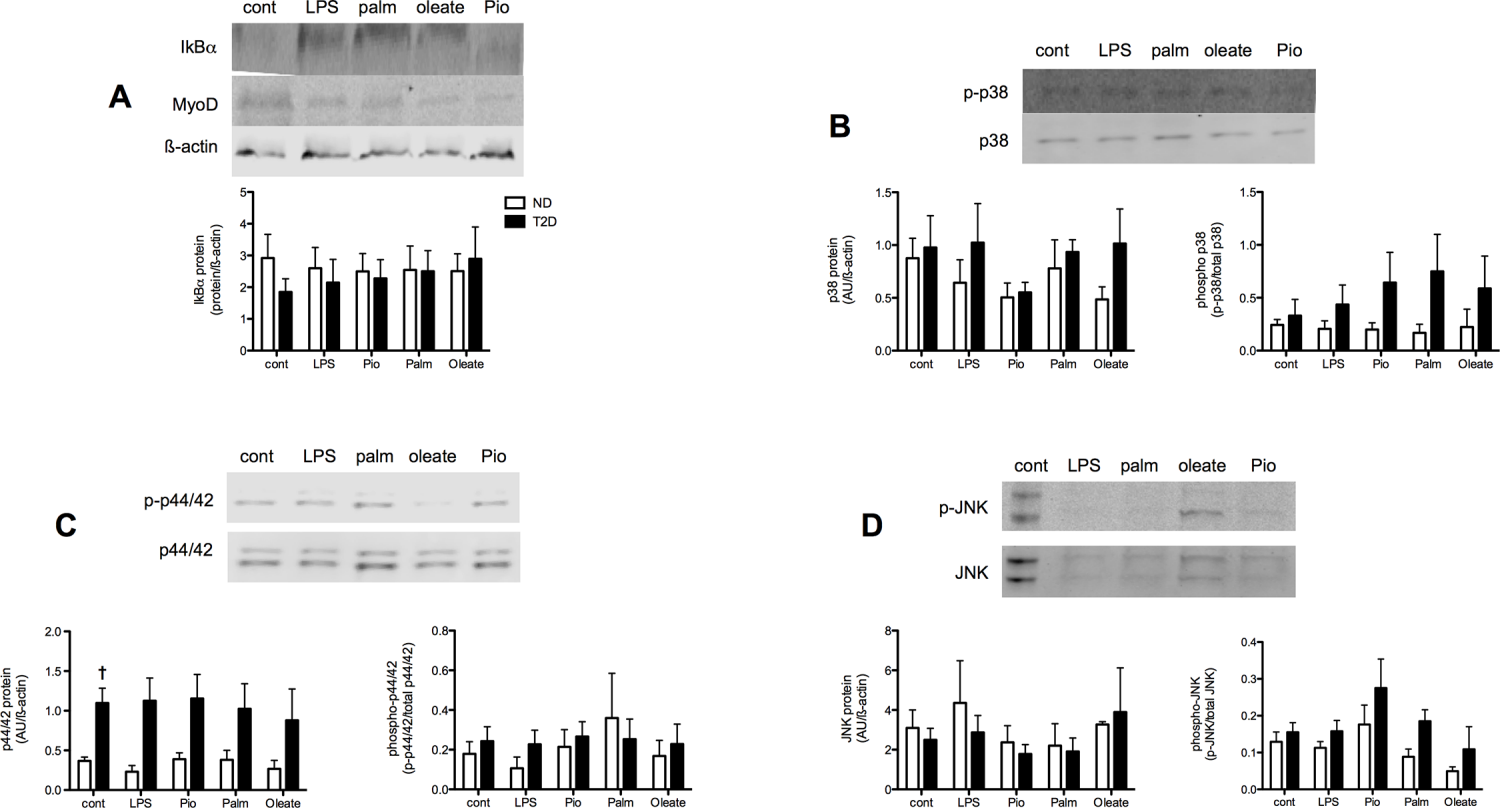
Regulation of inflammatory signaling in hSMC. ND (open bars) and T2D (solid bars) cells extracted after 48 hr treatment with LPS, Pioglitazone (Pio), palmitate or oleate. Results expressed relative to untreated control for each individual set of cells, Ave + SEM. (A) IkBa protein, n = 7–12 and 6–15 for ND and T2D, respectively. (B) Total and phospho-p38, n = 6–14 and 5–8. (C) Total and phospho-p44/42, n = 7–13 and 7–11. (D) Total and phospho-JNK, n = 9–12 and 3–8. * p<0.05 T2D vs ND. †p<0.05 T2D response vs ND response

Neither pro- (LPS) nor anti- (Pio) inflammatory treatments that induced changes in myokine secretion had consistent effects on IkBa expression in ND or T2D myotubes (Fig 5A). Treatment with palmitate and oleate also did not lead to consistent changes in IkBa protein expression. NFkB protein expression in both ND and T2D hSMCs was unaltered by any of the treatments (data not shown). None of these treatments displayed consistent or statistically significant effects on the extent of myotube differentiation, as indicated by expression of the myogenic determination factor MyoD (example in Fig 5A) (LPS—140 ± 34% of paired control, palm– 101 ± 8%, oleate– 88 ± 21%, pio– 95 ± 31%, n = 7–8).

There were tendencies for Pio(p = 0.08) and oleate (p = 0.057) to down regulate p38 expression in ND but not T2D myotubes (Fig 4B) (p = 0.091 comparing the responses of ND vs T2D cells). There was a weak tendency for LPS (p = 0.082) to increase p38 phosphorylation in T2D hSMC but not ND cells (p = 0.053 comparing the responses between the groups). None of the treatments led to consistent effects on either the protein expression or phosphorylation of p44/42 (Fig 5C) or JNK (Fig 5D).

## Discussion

The importance of skeletal muscle in locomotion and metabolism is undisputed. Over the last 10–15 years another function of skeletal muscle has been established, that of an endocrine tissue, secreting factors into the interstitial space and circulation [3]. Given their tissue of origin, such factors are termed “myokines”, analogous to the adipokines produced in adipose tissue [6]. Since skeletal muscle contains a number of different cell types in addition to myotubes that can also produce cytokines and chemokines, the question arises of what is needed to classify a factor as a myokine? A key feature of such a definition is that a myokine be secreted, placing importance on measuring release from muscle, in addition to monitoring changes in gene expression [6]. Furthermore, the site of myokine synthesis and secretion as specific to muscle should be verified by studying muscle cells [6].

For the latter reason rodent skeletal muscle cell lines have been useful in identifying myokines and exploring their regulation. For example, the expression of a number of myokines is modified during myogenesis in C2C12 cells [29]. However, rodent muscle cell models can provide limited insights into human pathophysiology. Fortunately, multiple investigators have shown that hSMCs proliferated and differentiated in vitro display many of the features of mature muscle [16]. Indeed, hSMCs obtained from T2D subjects retain many of the abnormal metabolic properties that their donors express in vivo. These include: Impaired basal and insulin-stimulated glucose uptake [17, 30], reduced glycogen synthesis [18], impaired fatty acid oxidation [19], and altered protein expression [31],

Under the criteria described above, IL6 was identified as early as 1994 as a myokine, constitutively secreted by human myotubes [32]. This was followed by the work of Pedersen and colleagues on the dynamic exercise-mediated regulation of IL6 synthesis and secretion [4]. Mirroring what is seen in vivo, production of multiple myokines by human myotubes have been reported to be regulated by differentiation [8], exercise [5] and electrical stimulation [9, 10]. Information regarding whether myokine secretion is altered in T2D is somewhat more limited. Novel information provided by the current report includes: 1) GROa, IL8, IL15 and follistatin are myokines whose secretion is higher in T2D, 2) secretion of IL1ß and VEGF is similar between ND and T2D myotubes, and, 3) IL10 secretion by resting myotubes is modest. Furthermore, the results indicate that INFg does not meet the criteria for a myokine, in agreement with the recent report of Brown *et al* [33]. Our current observation that TNFa secretion, while low, is elevated in T2D myotubes is confirmatory of our earlier results [13] and in agreement with Green *et al* [34] and Vandanmagsar *et al* at the level of gene expression [12]. Consistent with these results, induction of an insulin resistant state by TNFa treatment of hSMC from healthy individuals also resulted in increased TNFa production and secretion [11]. The picture regarding IL6 is more mixed. While we found IL6 secretion to be higher in T2D hSMC, as did Bouzakri *et al* after TNFa treatment of ND myotubes [11], others found no difference from ND [14, 35] or even decreased release [34]. The observations that MCP-1 mRNA is elevated in T2D hSMC [12, 36] and after TNFa treatment, and that secretion is also elevated in the later case [11], would be congruent with our results regarding MCP-1 release. Thus, our results that secretion of TNFa, IL6 and MCP-1 are all elevated in T2D myotubes are in agreement with the findings of others, combining observations at the level of gene and protein expression, to which we have added information about the secretion of GROa, IL8, IL15, IL1b, VEGF, IFNg and follistatin.

What might be the consequences of elevated secretion of selected myokines by T2D muscle? They could contribute, in part, to the T2D metabolic phenotype through autocrine effects. TNFa [11, 39], IL6 [4] IL8 [37], IL13 [38] and IL15 [37] have all been shown to modulate muscle glucose or FFA metabolism. However, these results are often seen experimentally using concentrations that are orders of magnitude higher than those produced by myotubes alone (Fig 1). This would be consistent with our observation of the failure of treatments that altered myokine secretion (LPS and palmitate) to induce consistent changes in glucose uptake or FFA oxidation (Fig 3). A similar finding was reported by Brown *et al*, who found that changes in cytokine gene expression following inhibition of p38 MAPK activity in T2D hSMC were not accompanied by alterations in insulin-stimulated glucose uptake [33]. It is possible that the high levels of the factors mentioned above that are required to influence metabolism in skeletal muscle would be attained in muscle tissue only after recruitment of inflammatory cells, which also secrete many of the same factors [6].

Interestingly, the three myokines of interest whose circulating levels were elevated in our cohort of T2D subjects, TNFa, GROa and follistatin (Table 1), were also secreted to a greater extent by T2D myotubes (Fig 1). Diabetes-related elevations in circulating TNFa [13, 39], GROa [40, 41], and follistatin [7, 42] have been reported previously. Given the quantities of GROa and follistatin that are secreted by myotubes (Fig 1), it is likely that skeletal muscle is a major contributor to their levels in the circulation; raising the possibility of paracrine end endocrine effects of these factors. However, recent evidence indicates that it is the liver that is the primary source of circulating follistatin [43]. The low amount of TNFa secreted by myotubes (Fig 1) [13] suggests that other tissues are the major determinants of circulating TNFa.

There is evidence for both increased [44–46] and unaltered [47] inflammation in skeletal muscle associated with obesity and the metabolic syndrome. However, the situation regarding T2D is mixed, with reports of both low macrophage content [48, 49] and elevated inflammatory gene expression [50–52] in skeletal muscle of obese T2D subjects. There are several reports that myotubes from obese T2D subjects display elevated NF-kB activity compared to cells from lean-healthy individuals [12, 34]. We observed no group or treatment differences in NFkB expression or phosphorylation, but did not measure activity directly. There was a recent report of increased p38 MAPK activity in T2D myotubes, which contributed to increased cytokine gene expression [53]. We found no differences in p38 phosphorylation. However, these autocrine effects may be somewhat limited, as sizeable changes in myokine secretion with LPS, palmitate or Pio treatment resulted in modest, or no, changes in IkBa content and the expression or activity of inflammatory signaling pathways. Just as for the effects on metabolism mentioned above, it may be the paracrine and endocrine actions of certain myokines to recruit infiltration of inflammatory cells into SkM that are more important in shaping the inflammatory tone of myotubes, rather than autocrine actions alone.

Two of the factors most abundantly over-secreted by T2D myotubes, GROa and IL8, have a number of common properties, including structure (the CXC class of chemokine), and receptors (CXCR1 and CXCR2). Circulating levels of both are elevated with obesity [54, 55], in obese T2D individuals [40, 41] and, perhaps paradoxically, after exercise [56, 57]. Shared actions of GROa and IL8 include neutrophil recruitment and regulation of angiogenesis [58]. Interestingly, the elevated secretion of IL15 and GROa by T2D myotubes, which display impaired fatty acid oxidation, would seem to be in conflict with studies in transgenic mice, where muscle-specific overexpression of IL15 [59] and CXCL1 [60] (the rodent homologue of GROa) each resulted in improved fat metabolism. Several reasons could account for these discrepancies. One could be species differences, as is highlighted by a recent report showing no difference in SkM interstitial IL15 content between lean and obese, non-diabetic, humans [61]. Another would be a limitation of the hSMC system, where only autocrine effects are possible, omitting potential paracrine and endocrine actions, including the contributions of other tissues, such as resident and recruited macrophages and neutrophils. Perhaps the most important of the contributions from other cells/tissues would be the enervation of muscle, a component missing from the system employed in the current report, as contractions induced or mimicked by electrical stimulation have been shown to have profound effects on myokine secretion [9, 10].

There are several other aspects of the current study that require consideration. One is the difference in average BMI between the two groups of subjects. While adjusting for BMI influenced differences in circulating TNFa and GROa levels, we found no statistically significant associations between donor BMI and myokine secretion from hSMC in our subjects. Given the multiple examples of a lack of congruence between mRNA and protein for secreted proteins [15], we chose to focus on the final stage, secretion itself. Furthermore, the current report looks at only a small number of potential myokines. The decision was made to take a targeted approach, focusing on a limited number of factors, including several that have been under study by others [11–14, 33–36], as well as factors for which there is evidence that they could possibly play roles in the diabetic phenotype.

In conclusion, human skeletal muscle cells maintained and differentiated in culture have proven to be valuable tools for studying metabolic regulation, as cells from T2D subjects retain many of the metabolic properties displayed in vivo. We, and others have described these behaviors as intrinsic to muscle in diabetes, since they persist even under controlled conditions, including normo-glycemia and -insulinemia. The finding that T2D hSMC are capable of creating a potentially pro-inflammatory environment, relative to ND cells, complicates that interpretation. Rather, the quantitative differences in myokine secretion between ND and T2D myotubes in the baseline state could contribute directly and/or indirectly to the well-established impairments in glucose and fatty acid metabolism in T2D muscle and other tissues. This could be due in part to modest autocrine effects, but also to possible paracrine (such as regulation of angiogenesis in SkM [62]) and endocrine (recruitment of inflammatory cells, regulation of insulin secretion [11]) actions. Thus, the aberrant secretory function of skeletal muscle in T2D could have an impact on multiple tissues, supporting full expression of the diabetic phenotype.
